# The Earlier Practice of Dentistry in Hawaii

**Published:** 1904-04

**Authors:** J. M. Whitney

**Affiliations:** Honolulu


### THE EARLIER PRACTICE OF DENTISTRY IN HAWAII.

BY DR. J. M. WHITNEY, HONOLULU.

Mr. President and Gentlemen of the Dental Association of Hawaii,—At this close of the pleasant relationship we have sustained during the past year, I wish to express my appreciation of the honor conferred upon me, in electing me the first president of this Association. I thank you for the kind assistance you have rendered, and am glad of the unanimity with which we have worked, and the sense of good fellowship which from the first we have enjoyed. I think, for our first year, we have reason to congratulate ourselves upon what has been accomplished. Besides becoming fully organized, we have had two excellent papers, with a full discussion of them. I feel sure the coming year will witness an even more active growth. Let us labor earnestly to induce all our members living in Honolulu to attend all our meetings, and try to make them feel that the success of the Association rests upon all alike.

As your oldest member, who has practised our profession upon these islands much the longest time, I have thought that a hasty review of some things I have seen and done may be interesting to you, my younger brothers.

At the time of my graduation from the Pennsylvania College of Dental Surgery, in 1868, there had been but about six hundred dental students who had previously graduated, and so we felt that practically the world lay before us.

In partnership with a friend and classmate, I had prepared and expected to make some city of France my future home. But unexpected occurrences prompted, and I was obliged to look for another field. While still undecided where to locate, an earnest invitation came to a friend of mine to come to Honolulu, as there was no dentist here at the time. My friend did not care to accept, and turned the invitation over to me. As soon as we could make our arrangements my wife and I were married and started upon what seemed a very long wedding-journey in August, 1869.

The Union Pacific Railroad had been opened less than a month when we crossed the continent. After leaving Omaha we found ourselves in a wild country indeed. Faro and other gambling games were at many stations placed by the side of the cars to catch the unwary, and in every car was a conspicuous notice: "Passengers are warned against playing three card monte or other games of chance with strangers. You will surely be robbed if you do."

In San Francisco I was strongly urged to stop or go to Oakland, where at that time there was not a dentist! But we continued our way, and reached Honolulu September 12, 1869, and received an immediate and hearty welcome. Dr. Mott-Smith, after a long and most successful practice, had given up the dental profession, and gone into government employ under the king, Kamehameha V. Much of Dr. Smith's practice fell to me, and, considering that his knowledge had been gathered many years before from one dental office, much of it was very commendable. He evidently knew little about the treatment of teeth with devitalized pulps, but his soft-gold fillings were above the average of his day, and his mechanical work was excellent. But the best thing he did for his people was strongly impressing them with the value of their teeth, and the necessity of frequent and continued watching and caring for them. The result was that when I came with more modern knowledge and methods, I found them eager to know the best methods of saving their own and their children's teeth. I think I am safe in saying that no man ever had quite so pleasant a practice as was mine in those early days. We all know that delightful sensation when we feel we have the full confidence of our patient, and are at full liberty to go forward and do what under the circumstances we consider the very best to be done, without consulting or considering cost. This was in the main my experience, and I enjoyed it to the full.

Allow me to tell you that I brought, in the way of preparation, to my field of work eighteen months instruction by one of the best operators I ever knew, and two years in a dental college, with two full courses in anatomy in Jefferson Medical College, Philadelphia. The germ theory of decay of teeth was, of course, then unknown. The chemical theory was then universally received. Cohesive gold had but recently been introduced, and all students were trained in the use of soft gold and tin, a legacy I have valued above price. The use of the rubber dam had not been introduced into the college curriculum, and all our longest and most difficult operations had to be performed with napkins without the use of the siphon. The careful work we had to do in this line was not lost to future usefulness.

I had brought some rubber dam with me, but it deteriorated so rapidly in this climate that I was generally unable to use it. We had no rubber dam clamps then, which increased the difficulty. I noticed among my Hawaiian patients who were awa users, that their mouths were excessively dry, and it occurred to me that I might make use of the awa to reach places before inaccessible. So in some cases where the rubber dam could not be held, and the mouth was very moist, I tried the experiment of having the patient chew a little awa for fifteen or twenty minutes before I began to operate. In most cases this was successful. However, very soon the rubber dam clamp was introduced, and the awa was of no further use.

We had been taught the destruction of the pulp and the treatment of pulp-canals, filling them with gold or gutta-percha, but as the germ theory was then unknown, much that we did was empirical. Nothing was then taught about pyorrhœa alveolaris, though I soon found thorough cleansing of the teeth and roots and treatment with oxychloride of zinc very helpful. About two years after commencing my practice here Dr. Riggs, of Hartford, Conn., wrote his first articles for the *Dental Cosmos* upon his treatment of this disease, and from that time to the present the mode of treatment has changed but little. Some two years after his writings appeared I had the privilege of spending a day with Dr. Riggs in his office, and through his courtesy and kindness I gathered much that has since been of great value. The present methods of crowning and bridge-work were unknown in our college work, and it must be within the memory of many of you present when they were first brought clearly before the profession.

The first dental machine was invented by a Dr. Green. He lived in Milwaukee, Wis., if my memory serves me right. This was pneumatic, and the burs and drills screwed into the hand-piece. I possessed the fourth one that crossed the Rocky Mountains, and found it of very great help. After some two or three years that was superseded by the present machine invented by Dr. Morrison, of St. Louis, which was the same as the one now in use excepting the great improvement given it by the steel cable, an invention of Mr. A. H. Kennedy, brother of Mrs. W. A. Bowen of this city, a young man who lived in a town adjoining the one in which I spent my boyhood. I well remember the interest excited among the boys of my town when it was told that young Kennedy had invented a machine for conveying power, which was first used for shearing sheep, and many went to see it work, but I did not happen to be among the number. He sold his patent to S. S. White for the small sum of three thousand dollars, and probably he made a million on it, as there is hardly any place where power is to be transferred where it is not used. Of course this put the Morrison machine in much the same place as the Morrison had put the Green machine.

Through the training of my preceptor, whose superior I never saw in filling with gold, and my teaching in the dental college under Dr. James Truman, I became deeply impressed with the superiority of gold for the filling of teeth above every and all substitutes. From the first to the present, with very few exceptions, unless the question of expense prevented, it has been used, and my thirty-five years' experience has more than proved to my mind that, without regard to age except in the anterior teeth of the young, especially of Hawaiians, gold for durability and cheapness in the end far outranks any and all other material. Rarely a week or day passes that I do not see gold fillings in occlusal or approximate surfaces that have been doing perfect service for ten, twenty, thirty, or more years. Fillings of recent date, made with all the instruction given by such men as Dr. Black and a host of other gold workers, should prove much superior to those of early days. Some years ago, before preparing a paper for the California State Dental Association on the value of gold for fillings, I put my ledger into the hands of a professional book-keeper, in order to eliminate the personal element, and he chose the first twenty-one names that I had had upon my books for twenty-one years, followed each filling through the twenty-one years, and in summing up the results found but five per cent. had required refilling within that time. Oxy-phosphate was then unknown to the profession at large if at all. Oxychlorate was known but rarely used. As it was then made it was of small value.

Silver alloys have been greatly improved since I began practice. Arrington's was probably as good as any, but then as now they proved a very uncertain material upon which to rely. We were carefully trained in regard to cleanliness of all instruments to be used in the mouth, though without our present knowledge of germs and their influence. I may say, however, that in nearly forty years of life in a dental office, under all the many conditions I have seen, I have never met a single case that showed that disease had been communicated by dental instruments in the hand of a regular dentist. I do not mention this to detract from the great importance of the most scrupulous care with our instruments, but to show that there is less danger than some would have us believe who write scare articles for our journals. We cannot be too careful, but to make capital by working upon the fears of our timid patients in this direction is not only unprofessional, but has in it all the elements of the charlatan. Do unto others as you would be done by, use upon others any instrument or appliance that you would be willing under the same conditions to have used upon yourselves, is to me a good and safe rule, and make no show about it.

I used to battle with my preceptor about his wholesale extraction of teeth, but he was but following the peculiar conditions of the times. Vulcanizable rubber had then just been discovered. Gold and silver plate, with their difficult working and expense, had up to this time held back the untrained and ignorant from entering the dental profession. There was no law regulating the practice of dentistry. Any one by paying a hundred or two dollars could go into a dental office, in a couple of weeks learn to make a set of teeth on rubber base, and the week after hang out his sign as a dentist. The people, then as now, expecting to get something for nothing, would flock to his rooms. He knew nothing but to drag out their teeth and put in their place his miserable substitute for as miserable a pittance, and thus began the great destruction of the people's teeth and the beginning of the sad condition we see to-day in the mouths of their children. For I think it is the testimony of every observant dentist, that he has never seen in the mouth of a descendant a normal condition of the teeth, whose mother has before his birth lost her full upper or lower set or both.

When I first came to my practice in Honolulu it was the custom for the physicians to give instructions to the dentist what to do. This I resented with considerable spirit, for, as I said to them, "I have spent as many years in preparing for my specialty as you did for your general practice, and under as severe discipline, and it is but common sense that I should know more about it than you do who did not probably give it an hour of time in your full course." I had so much of this to contend with that I resolved to see for myself the foundation upon which they built their sense of such superior knowledge. I at once put every spare moment into study for a medical course, and, returning to Ohio, matriculated and graduated from one of the best medical schools in that State. I have to state that in the six months' course but a part of one hour lecture was given to the special diseases of the teeth and mouth. Doubtless that same school would now do much better by its graduates. It is needless to say that when I returned with my medical degree, I never heard further from my medical *confrères,* but was allowed to save teeth that I thought best to preserve without their contention.

The whole subject of antiseptic mouth-washes has grown up since my graduation—I am not quite sure but to the serious injury of the mouth and teeth. There is no question about the great care which should be given to cleansing the teeth as thoroughly as possible with brush and silk, using, if necessary, a carefully prepared tooth-powder once a day at night. But to be constantly washing the mouth at all times of the day with strong, pungent aseptic mouth-washes seems to me to weaken and put to sleep nature, which, left to herself, would prepare her battalions, and with her guards ever out would seek the most vulnerable points of her enemies, and thus be able to fight them much more successfully than would be done by foreign help.

I cannot close this reminiscent paper without saying something about our profession, and the inheritance with which you younger men have entered. After I had returned from service in our Civil War, and had decided to study dentistry, several of my influential friends were greatly opposed to it, and said, "You are too much of a man to go into that miserable charlatan kind of work. It is no place for you; you can find a better calling." I mention this simply to show the feeling existing at that time against the profession. And can it be said to have quite disappeared at the present time? After my graduation I went to visit a dear old friend whose life was among the upper classes. Her daughter told her I had graduated a doctor. They did not dare tell her I was to be a dentist, for it would greatly shock her and cause her to commiserate me instead of congratulating me as she did. To the day of her death she thought of me as a physician, and was happy in the thought. The first instance was in Ohio, the second in Vermont, so you see the same feeling prevailed east and west. Physicians would not recognize our title of D.D.S., and if they called us doctor it was with much the same spirit as they might give the title to an apothecary. In a measure we deserved some of the opprobrium, for many graduated were without much if any preparatory study, and thus were at great disadvantage with their much more highly cultured brothers, and the great majority gave themselves the title of doctor, and of course such could have no standing with the regulars.

But to you young men this is ancient history. You find yourselves in a learned profession with your degree recognized by the International Medical Association, the highest authority, as on a par with the degree of M.D. You have reason to feel but little of that slight which the older members had to carry. We to-day ought to give thanks and reverence to those who so bravely and persistently fought our battles before legislatures and courts and the more trying and cynical great meetings of scientific and medical men, and greater still by their true lives and great usefulness before the people. We cannot stop more than to mention the names of Dr. Harris, Dr. Taft, Dr. Tucker, Dr. Atkinson, and Dr. Barrett, great men of our profession who have passed on before. And the host of such names as Dr. Truman and Dr. Brophy and Dr. Black and Dr. Harlan and Dr. Kirk and Dr. Johnson, who are still in the great work of bringing our profession to the forefront. It is no mere boast to say that our profession does more than any or all others in assuring comfort and happiness to mankind. Therefore let us take great pride in it, and be loyal to its highest ideals. Let us feel by our true lives and earnest work that we are the peers of any who call upon us for our services, and let us see to it that no man nor class of men shall exceed us in honest, earnest and intelligent endeavors to minister to the well-being of our fellows.

